# Comparative Gene Expression Analysis of Lymphocytes Treated with Exosomes Derived from Ovarian Cancer and Ovarian Cysts

**DOI:** 10.3389/fimmu.2017.00607

**Published:** 2017-06-01

**Authors:** Yujuan Li, Yang Yang, Aiwei Xiong, Xiaoqin Wu, Jingyan Xie, Suping Han, Shuli Zhao

**Affiliations:** ^1^Nanjing First Hospital, Nanjing Medical University, Nanjing, China; ^2^State Key Laboratory of Reproductive Medicine, Nanjing Medical University, Nanjing, China

**Keywords:** exosomes, ovarian cancer, ovarian cysts, lymphocytes, tumor microenvironment

## Abstract

Cancer cells employ many strategies to evade immune defense and to facilitate tumor growth and angiogenesis. As a novel mode of intercellular communication, cancer-derived exosomes contribute to the recruitment and mediation of lymphocytes within the tumor environment. However, the mechanisms and key molecules mediating the effect of exosomes on lymphocytes are unclear. We treated healthy peripheral blood lymphocytes with exosomes from ovarian cancer and ovarian cysts and screened for differentially expressed genes using the RT^2^ Profiler Cancer Inflammation and Immunity Crosstalk PCR Array. A total of 26 upregulated genes (mainly pro-inflammatory genes and immunostimulatory and immunosuppressive factor) and two downregulated genes (antigen presentation HLA-A/B) were identified. Western blotting using lymphocytes from malignant ascites and peritoneal washings of benign ovarian cysts suggested that the interferon and NF-κB signaling pathway were involved in the immune regulation of malignant exosomes. Out of 28 differentially expressed genes detected using the array, 11 were validated by real-time PCR using lymphocytes within ovarian cancer (*n* = 27) and ovarian cyst (*n* = 9) environments. In conclusion, our findings indicate that malignant cells secrete exosomes in the tumor microenvironment to recruit lymphocytes in order to suppress antitumor immunity (IL10, Foxp3, and HLA-A/B) and enhance tumor invasion, angiogenesis, and dissemination of proinflammatory cytokines (such as IL6 and VEGFA) *via* the interferon and NF-κB signaling pathways. These results clarify lymphocyte-cancer cell cross talk *via* exosomes and may facilitate the development of effective immunotherapeutic strategies for ovarian cancer.

## Introduction

Ovarian cancer is the fourth most common female cancer worldwide and has the highest mortality rate of all gynecologic cancers in China (1, 2). Many studies have demonstrated that the tumor cell microenvironment plays an important role in ovarian cancer progression (3–5). However, the precise roles of microenvironment–tumor interactions are unclear, including the key substances in the microenvironment and the mechanisms that trigger tumor development, angiogenesis, and metastasis. Recently, many studies have reported that exosomes in the microenvironment, which are secreted by most normal and malignant cells, play critical roles in the occurrence and development of cancer (6–9).

As a novel mode of intercellular communication, exosomes can shuttle between cells and transmit signals by transporting various bioactive proteins and nucleic acids (e.g., DNA, mRNA, and miRNA) (10, 11). Andre et al. reported that exosomes isolated from ovarian cancer ascites contain antigen-presenting molecules, tetraspanins (CD81), and tumor antigens (Her2/Neu, Mart1, TRP, and gp100) (12). Growth factors, including tumor necrosis factor-alpha (TNF-α), epidermal growth factor, and fibroblast growth factor, as well as mRNAs and miRNAs have been detected in exosomes (13). To date, 4,563 proteins, 1,639 mRNAs, and 764 miRNAs have been found in exosomes (14).

As a permissive reactive tumor–host microenvironment, malignant ascites provide sustenance for the growth and progression of ovarian cancer, including cytokines, chemokines, growth factors, and so on (15, 16). Our previous studies have demonstrated that immune cell subsets and the expression levels of miRNAs (17) that regulate the differentiation and development of dendritic cells in ascites differ from those in the peripheral blood of ovarian cancer patients (18), and these differences can result in the immune escape of tumor cells. Similarly, the same amenities in ascites could help tumor cells evade host immunosurveillance, enabling unlimited tumor cell growth (19, 20). However, the causal factors in ascites and specific mechanisms are poorly understood. Exosomes may play an important role and accordingly have been a focus of recent research (21, 22). In this study, the mRNA profiles in peripheral blood lymphocytes (PBLs) stimulated by exosomes derived from benign or malignant ascites were examined using the RT^2^ Profiler PCR Array and the key effectors of exosomes with respect to the immune system were explored.

## Materials and Methods

### Subjects

In total, six epithelial ovarian cancer (EOC) patients and six benign ovarian cyst patients were recruited from the Department of Gynecology at Nanjing First Hospital, Nanjing Medical University (Nanjing, China). The diagnosis of EOC and benign ovarian cysts were in accordance with recommended criteria proposed by the World Health Organization in 2004. In addition, five age- and gender-matched healthy volunteers were recruited as healthy control. Sterile ascites from EOC patients or peritoneal washings from ovarian cysts were collected in sterilized 50-mL centrifuge tubes during surgery prior to therapeutic treatment. For isolation of peripheral lymphocytes, fasting venous blood samples (10 mL) from each healthy volunteer were collected by vein puncture using. All research involving human participants was conducted with approval from the Ethics Committee of Nanjing Medical University. Written informed consent was obtained from all participants. None of the patients were treated with preoperative chemotherapy or radiation.

### Isolation and Quantification of Exosomes

Exosomes from all samples of malignant ascites or peritoneal washings were prepared using Total Exosome Isolation (from body fluids) (Catalog Number: 4484453, Invitrogen Inc., Carlsbad, CA, USA) according to the manufacturer’s instructions, with some modifications (23). Briefly, all samples in sterilized 50-mL centrifuge tubes were centrifuged at 2,000 × *g* for 30 min to remove floating cells and debris. Then, supernatants were thoroughly mixed with 0.5 volumes of Total Exosome Isolation Reagent (from other body fluids) by pipetting repeatedly until the solution was homogenous, and samples were incubated at room temperature for 30 min. After incubation, the mixed solutions were centrifuged at 10,000 × *g* at room temperature for 10 min (XE-90; Beckman Coulter, Brea, CA, USA). The exosome pellets were washed and re-suspended in 100 µL of phosphate-buffered saline (PBS) for quantification using a CD63 exoELISA (catalog# EXOEL-CD63A-1; System Biosciences, Mountain View, CA, USA) according to the manufacturer’s instructions (24). Finally, the purified exosomes were re-suspended in PBS at a concentration of ~10^10^ exosome particles per milliliter and stored at −80°C until use.

### Electron Microscopy

Transmission electron microscopy was used to evaluate the morphology of isolated exosomes (25). Briefly, the exosome pellets were fixed in 5% glutaraldehyde for 2 h at 4°C, and were washed with phosphoric acid buffer four times for 15 min each. After 2 h of fixation by 1% osmic acid at 4°C, the adsorbed exosomes were washed with phosphoric acid buffer two times for 5 min each. Then, the prepared exosomes were stained with 2% uranyl acetate for 2 h and dehydrated in acetone. After they were embedded in embedding agent, the exosomes were sectioned at a thickness of 50–80 nm on an ultramicrotome. Finally, the prepared exosomes were observed under a transmission electron microscope (JEM-1010; JEOL, Tokyo, Japan) at 80.0 kV, and images were captured using a digital camera.

### Coincubation of PBLs with Exosomes

Peripheral blood lymphocytes from five aged-matched healthy female volunteers were isolated using lymphocyte separation medium (TBDscience, Tianjin, China) density gradient centrifugation according to the manufacturer’s instructions. Then, PBLs were purified using anti-CD45-PE antibody fluorescence-activated cell sorting performed on a Coulter Epics Elite ESP Flow Cytometer (Coulter, Brea, CA, USA). Subsequently, sorted PBLs were cultured in 6-well plates at 1 × 10^6^ cells/well in triplicate using the AIM-V-free serum medium CTS (Gibco, Waltham, MA, USA). Isolated exosomes (three malignant exosomes or three benign exosomes, 1 × 10^9^ exosome particles per milliter concentration) were added to each well, respectively. The cells were cultured for 48 h at 37°C. Following extensive washing in PBS to remove exosomes, all PBLs in every group were pooled to isolate RNA.

### RNA Extraction and cDNA Preparation

After coincubation with different exosomes, PBLs were collected to extract total RNA using the E.Z.N.A.^®^ Total RNA Kit II (OMEGA, Biel, Switzerland, USA) according to the manufacturer’s instructions. Next, the cDNA was synthesized using the RT^2^ First Strand Kit (Qiagen, Valencia, CA, USA) following the manufacturer’s instructions.

### RT^2^ Profiler™ PCR Array

Eighty-four genes or biological pathways involved in mediating communication between tumor cells and the cellular mediators of inflammation and immunity were analyzed using the RT^2^ Profiler Cancer Inflammation and Immunity Crosstalk PCR Array (PAHS-181Z; SABiosciences, Frederick, MD, USA) (26). According to the manufacturer’s protocol, real-time PCR was performed using RT^2^ Profiler PCR Arrays in combination with RT2 SYBR Green/ROX PCR Master Mix (Qiagen). A 102-µL cDNA synthesis reaction volume was mixed with 2 × RT2 SYBR Green Mastermix and RNase-free water to obtain a total volume of 2,700 µL. Subsequently, 25 µL of the PCR component mix was placed into each well of the PCR array (a 96-well array). The three steps of the cycling program were 95°C for 10 min for 1 cycle, followed by 40 cycles of 95°C for 15 s and 60°C for 60 s. This process was repeated for 40 cycles using the ABI-7500 (Applied Biosystems, Waltham, MA, USA). The expression levels were quantified relative to the values obtained for housekeeping genes (*ACTB, B2M, GAPDH, HPRT1*, and *RPLP0*). Data analyses were performed using web-based analysis software (http://pcrdataanalysis.sabiosciences.com/pcr/arrayanalysis.php).

### Statistical Analyses

GraphPad Prism5 (San Diego, CA, USA) was used to analyze the clinical data. A *t*-test or two-way ANOVA followed by Tukey’s *post hoc* test was used to determine significant differences between two or more groups, respectively. A *p*-value of less than 0.05 was considered significant.

## Results

### Isolation and Identification of Exosomes

**Figure 1:**
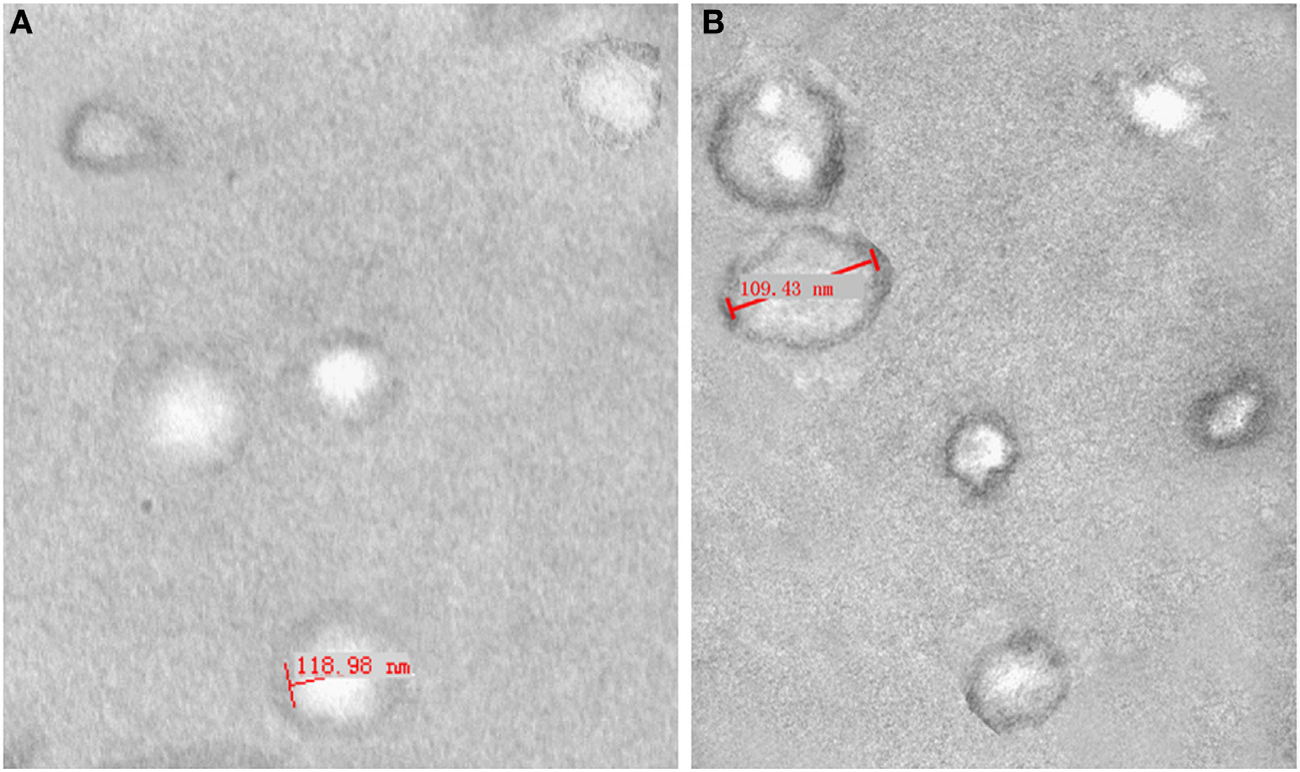
**Electron micrograph analyses of exosomes from peritoneal washings (A) and malignant ascites (B)**. Structural analysis of exosomes shows the presence of vesicles in the size range of 40–120 nm.

We assembled ascites or peritoneal washings from three ovarian cancer patients and three benign ovarian cyst patients. The isolated exosomes were identified under a transmission electron microscope at 80.0 kV and ranged from 50 to 100 nm (Figure 1). The expression of the tetraspanin protein CD63 was detected by ELISA to determine the concentration of purified exosomes re-suspended in PBS, i.e., 1 × 10^10^ exosome particles per milliliters. The solutions were stored at −80°C until use.

### Array Analysis

A multiphase case-control study was used to examine differences in the mRNA expression levels of immunity-related genes among healthy PBLs treated with different exosomes. The PBLs were collected from five matched healthy individuals and were pooled to eliminate individual differences. A total of 84 key genes involved in mediating the communication between tumor cells and the cellular mediators of inflammation and immunity were screened using the Human Cancer Inflammation and Immunity Crosstalk RT^2^ Profiler PCR Array in PBLs stimulated by malignant and benign exosomes. Based on positive PCR controls and reverse transcription controls, this profile had high reproducibility and efficiency according to the RT^2^ Profiler PCR Array Data Analysis.

**Figure 2:**
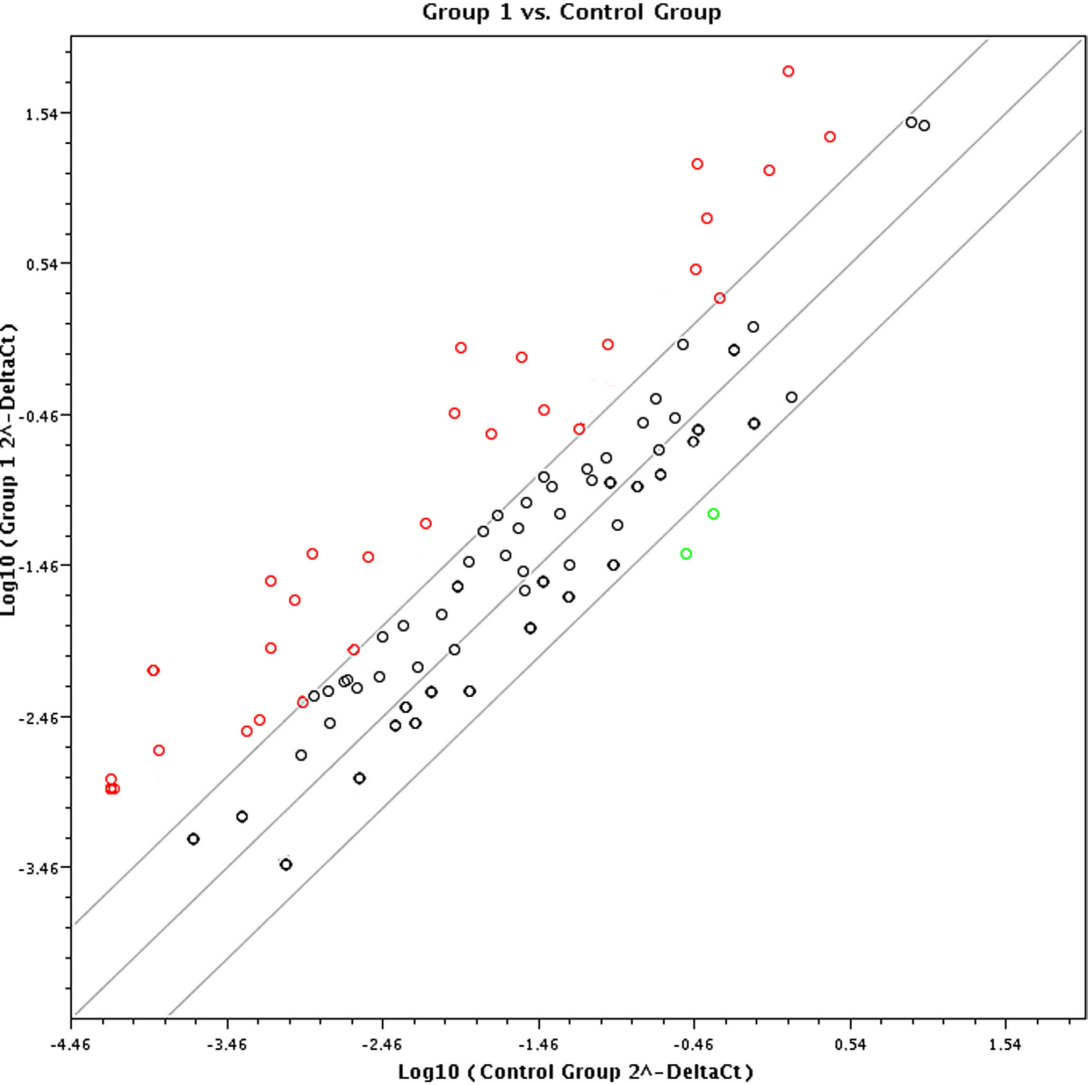
**Screening using the RT^2^ Profiler Cancer Inflammation and Immunity Crosstalk PCR Array (PAHS-181Z)**. Eighty-four cancer-related genes were analyzed using the RT^2^ Profiler™ PCR Array (*n* = 3 per group). Finally, 28 genes changed significantly between the malignant exosome group (group 1) and the benign ascite exosome group (control group).

In terms of the sensitivity and accuracy, the cutoff fold change (2^−ΔΔCt^) was set at greater than 5.0 or less than −4.0. Genes that met the two criteria (average threshold cycle <30 and the fold-change cutoff between the malignant exosome group and benign exosome group) were selected for further qRT-PCR validation using lymphocytes from malignant ascites of EOC patients and from peritoneal washings of benign ovarian cyst patients. We found that 26 genes were overexpressed, whereas only two genes were downregulated in the malignant exosome group compared to the benign ascite exosome group (Figure 2; Table 1, *p* < 0.05).

**Table 1:** **Profiler PCR array results for exosome-treated peripheral blood lymphocytes (malignant group vs. benign group)**.

Refseq	Gene	Fold change	*p*-Value	Description	Gname
NM_002982	CCL2	10.613	0.026	Chemokine (C-C motif) ligand 2	GDCF-2/HC11/HSMCR30/MCAF/MCP-1/MCP1/SCYA2/SMC-CF
NM_002984	CCL4	46.842	0.047	Chemokine (C-C motif) ligand 4	ACT2/AT744.1/G-26/HC21/LAG-1/LAG1/MIP-1-beta/MIP1B/MIP1B1/SCYA2/SCYA4
NM_002985	CCL5	9.331	0.031	Chemokine (C-C motif) ligand 5	D17S136E/RANTES/SCYA5/SIS-delta/SISd/TCP228/eoCP
NM_002988	CCL18	34.269	0.013	Chemokine (C-C motif) ligand 18	AMAC-1/AMAC1/CKb7/DC-CK1/DCCK1/MIP-4/PARC/SCYA18
NM_004591	CCL20	5.658	0.021	Chemokine (C-C motif) ligand 20	CKb4/Exodus/LARC/MIP-3-alpha/MIP-3a/MIP3A/SCYA20/ST38
NM_148672	CCL28	10.965	0.015	Chemokine (C-C motif) ligand 28	CCK1/MEC/SCYA28
NM_001511	CXCL1	35.459	0.007	Chemokine (C-X-C motif) ligand 1	FSP/GRO1/GROa/MGSA/MGSA-a/NAP-3/SCYB1
NM_002089	CXCL2	15.039	0.033	Chemokine (C-X-C motif) ligand 2	CINC-2a/GRO2/GROb/MGSA-b/MIP-2a/MIP2/MIP2A/SCYB2
NM_002994	CXCL5	6.084	0.015	Chemokine (C-X-C motif) ligand 5	ENA-78/SCYB5
NM_000584	CXCL8	16.523	0.039	Interleukin 8	GCP-1/GCP1/IL8/LECT/LUCT/LYNAP/MDNCF/MONAP/NAF/NAP-1/NAP1
NM_001565	CXCL10	11.039	0.031	Chemokine (C-X-C motif) ligand 10	C7/IFI10/INP10/IP-10/SCYB10/crg-2/gIP-10/mob-1
NM_005409	CXCL11	10.127	0.030	Chemokine (C-X-C motif) ligand 11	H174/I-TAC/IP-9/IP9/SCYB11/SCYB9B/b-R1
NM_000757	CSF1	5.473	0.023	Colony stimulating factor 1 (macrophage)	CSF-1/MCSF
NM_014009	FOXP3	7.014	0.047	Forkhead box P3	AIID/DIETER/IPEX/JM2/PIDX/XPID
NM_002053	GBP1	8.952	0.047	Guanylate binding protein 1, interferon-inducible	–
NM_004131	GZMB	13.799	0.032	Granzyme B (granzyme 2, cytotoxic T-lymphocyte-associated serine esterase 1)	CCPI/CGL-1/CGL1/CSP-B/CSPB/CTLA1/CTSGL1/HLP/SECT
NM_000619	IFNG	43.678	0.010	Interferon, gamma	IFG/IFI
NM_000576	IL1B	8.323	0.024	Interleukin 1, beta	IL-1/IL1-BETA/IL1F2
NM_000586	IL2	21.128	0.040	Interleukin 2	IL-2/TCGF/lymphokine
NM_000600	IL6	88.217	0.001	Interleukin 6 (interferon, beta 2)	BSF2/HGF/HSF/IFNB2/IL-6
NM_000882	IL12A	10.592	0.010	Interleukin 12A	CLMF/IL-12A/NFSK/NKSF1/P35
NM_000585	IL15	31.130	0.012	Interleukin 15	IL-15
NM_000247	MICA	13.941	0.031	MHC class I polypeptide-related sequence A	MIC-A/PERB11.1
NM_000582	SPP1	9.988	0.031	Secreted phosphoprotein 1	BNSP/BSPI/ETA-1/OPN
NM_003150	STAT3	8.592	0.033	Signal transducer and activator of transcription 3	ADMIO/APRF/HIES
NM_003376	VEGFA	11.365	0.037	Vascular endothelial growth factor A	MVCD1/VEGF/VPF
NM_002116	HLA-A	−5.914	0.034	Major histocompatibility complex, class I, A	HLAA
NM_005514	HLA-B	−7.404	0.012	Major histocompatibility complex, class I, B	AS/HLAB/SPDA1

### Cancer Immunity Pathway Analysis

The differentially expressed mRNAs in PBLs, which resulted from stimulation by malignant exosomes or benign exosomes, were mainly genes encoding for chemokines (C-C motif ligands and C-X-C motif ligands), interleukins, growth factors, and antigen presentation (Table 2). These overexpressed molecules were involved in immunostimulation, pro-inflammation, and immunosuppression in anticancer immunity. Acting as chemoattractants that guide cell migration, most chemokines (6/8 CC motif ligands and 5/7 CXC motif ligands) were more highly expressed after treatment with malignant exosomes than benign exosomes, suggesting that exosomes from cancer cells participate in immune surveillance, angiogenesis, and metastasis *via* the regulation of chemokine expression in PBLs.

**Table 2:** **Classification of differentially expressed genes according to their function**.

Immune and inflammatory responses		Number/total
Immunostimulatory factors	IFNG, IL2, IL12A, IL15	4/6
Immunosuppressive factors	CXCL5, VEGFA	2/16
Pro-inflammatory genes	CCL2 (MCP-1), CCL20 (MIP-3A), IFNG, IL1A, IL1B, IL2, IL6, IL12A, VEGFA	9/15
Enzymatic modulators of immunity	GZMB	1/6
**Chemokines**		
C-C motif ligand	CCL2 (MCP-1), CCL4 (MIP-1B), CCL5 (RANTES), CCL18 (PARC), CCL20 (MIP-3A), CCL28	6/8
C-X-C motif ligand	CXCL1, CXCL2, CXCL5, CXCL10 (IP-10), CXCL11 (I-TAC, IP-9)	5/7
Interleukins	IL1B, IL2, IL6, IL12A, IL15	5/13
Growth factors	CSF1 (MCSF), VEGFA	2/8
Antigen presentation	HLA-A, HLA-B, MICA	3/5

**Figure 3:**
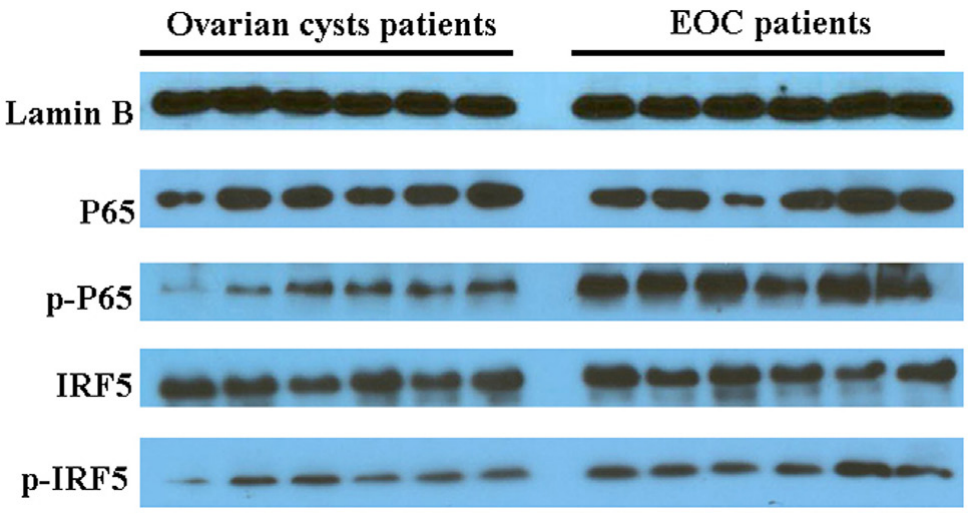
**Western blotting assays were used to analyze differences in signaling pathway activity between lymphocytes in malignant ascites of six epithelial ovarian cancer (EOC) patients and peritoneal washings of six benign ovarian cyst patients**. The phosphorylation levels of IRF3 and p65 in the nucleus of lymphocytes from malignant ascites were higher than those from benign ascites.

In an analysis of the roles of the differentially expressed genes in signal transduction, we found that malignant exosomes were involved in the tumor immune process mainly *via* the interferon signaling pathway, STAT signaling pathway, and NF-κB signaling pathway (Table 3). To confirm this, signaling pathway activity was examined in lymphocytes from malignant ascites of six EOC patients and peritoneal washings of six benign ovarian cyst patients by western blotting (Figure 3). We observed no differences in the phosphorylation levels of IRF5/7/9 and STAT1/2/3/5/6 in the nucleus. However, the phosphorylation levels of IRF3 and p65 in the nuclei of lymphocytes from malignant ascites were higher than those from benign ascites, suggesting that the interferon and NF-κB signaling pathways are involved in the immune regulation of malignant exosomes.

**Table 3:** **Classification of differentially expressed genes according to signal transduction pathway**.

Signal transduction pathway		Proportion
Interferon signaling	GBP1, IFNG, IL6	3/4
Interferon-responsive genes	CCL2 (MCP-1), CCL5 (RANTES), CXCL10 (IP-10), GBP1	4/10
NFκB targets	CCL2 (MCP-1), CCL5 (RANTES), CSF1 (MCSF), IFNG	4/9
STAT targets	CCL2 (MCP-1), CCL4 (MIP-1B), CCL5 (RANTES), CSF1 (MCSF), CXCL10 (IP-10), CXCL11 (I-TAC, IP-9), IL1B, IL6	8/17
Transcription factors	FOXP3, STAT3	2/8

### Validation of Lymphocyte Gene Expression in the Disease Microenvironment

**Figure 4:**
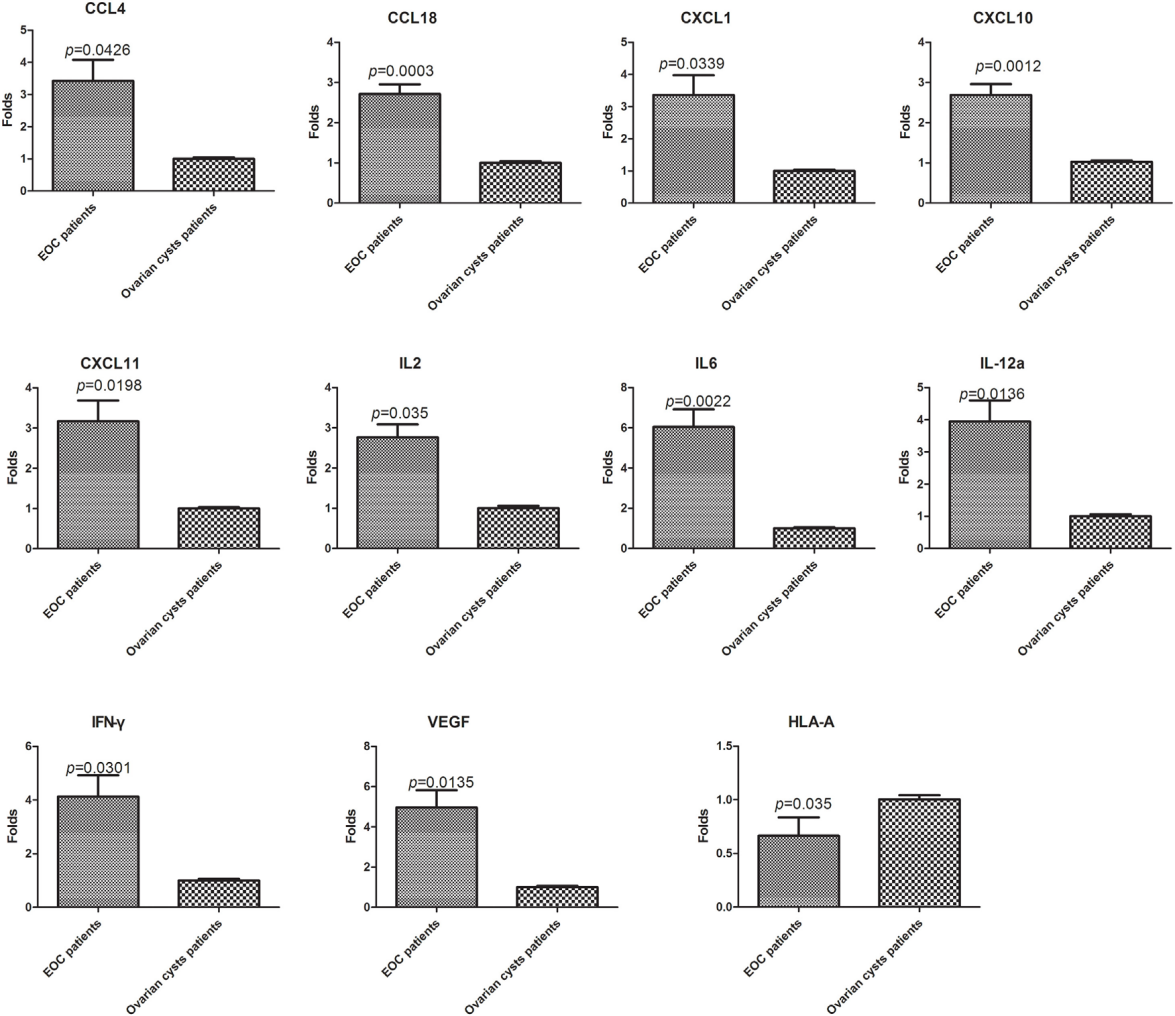
**Validation of the PCR screening results**. The 28 genes identified using the RT^2^ Profiler™ PCR array were validated using real-time PCR in lymphocytes from a larger sample of patients [27 epithelial ovarian cancer (EOC) patients and nine benign ovarian cyst patients]. Eleven genes showed significant differences (*p* < 0.05, *t*-test).

The 28 genes that were differentially expressed in normal PBLs treated with malignant or benign exosomes according to the array analysis were individually validated in lymphocytes from malignant ascites of 27 EOC patients or peritoneal washings of nine benign ovarian cyst patients by real-time PCR analysis. Regardless of clinicopathological characteristics, significant expression differences were observed, including 10 upregulated genes (*CCL4/18, CXCL1/10/11, IFN*γ, *IL2/6/12A, VEGFA*) and 1 downregulated gene (*HLA-A*) in lymphocytes from malignant ascites (Figure 4).

## Discussion

Favorable accessibility for initial cytoreductive surgery and sensitivity to chemotherapy can increase the cure rates for ovarian cancer. However, the immune system of patients often have a substantial negative impact on the clinical course of the disease and curative effects (27, 28). Emerging evidence has indicated that antitumor immunity is often negated by immune suppression mechanisms in the tumor microenvironment, and cancer-derived exosomes play a significant role in the recruitment and reprogramming of constituents in the tumor environment (29). Unlike healthy cells, cancer cells can secrete excess exosomes, and more importantly, they express distinct microRNAs with biological activity (30). Here, we used the commercially available RT^2^ Profiler™ Cancer Inflammation and Immunity Crosstalk PCR Array to identify useful molecular biomarkers and to clarify the pathway-based immunological system by evaluating differences in gene expression between PBLs treated with exosomes derived from benign and malignant ascites of ovarian cancer patients.

Exosomes are secreted by most cells to various bodily fluids, including the urine, blood, and cerebrospinal fluid, and actively participate in communication between cells *via* the transfer of bioactive proteins and nucleic acids (31). In this study, we used AIM-V-free serum to avoid interference caused by exosomes in fetal bovine serum. Currently, exosome research is limited by the lack of reliable and stable techniques to quantify and characterize exosomes. By detecting the CD63-specific antigen on exosomes, we standardized the stimulation of exosomes to fully explore differentially expressed genes in PBLs according to the biological properties and compositions of benign and malignant exosomes. Owing to limitations in available techniques, however, the purity of exosomes was not verified in this study. Accordingly, there may be trace amounts of other extracellular vesicles in the exosome samples.

Normalization strategies are critical for the success of mRNA expression studies. The combination of *ACTB, B2M, GAPDH, HPRT1*, and *RPLP0* were used to normalize mRNA expression in this study based on the instructions provided by the array manufacturer. However, the microarray results suggested that the mRNA expression of two common internal reference genes were differentially expressed (*GAPDH*, 2.2-fold; *ACTB*, −1.64-fold). Accordingly, the fold change cutoff was set at greater than 5.0 or less than −4.0 to improve the sensitivity and accuracy.

High-quality exosomes isolation from complex biological fluids is a critical step for downstream functional analysis and therapeutic applications. To this end, numerous protocols and commercially available reagents have been designed to purify exosomes from various samples. However, as far as we know, to date no perfect method for exosomes isolation has been identified to isolate all exosomes from 30 to 150 nm without any impurities. Our study is also limited by the methods used in this study that may also precipitate non-exosome debris in urine samples (32). The gene expression of PBLs resulted from exosomes could be interfered by the debris. So, we eventually detected 26 upregulated genes and two downregulated genes in the malignant treatment group compared to the benign treatment group. These 28 genes were individually validated in the lymphocytes of ovarian cancer ascites and peritoneal washings of ovarian cysts by real-time-PCR analysis. Eleven out of these 28 genes showed significant differential expression between cancer ascites and ovarian cysts (*CCL4/18, CXCL1/10/11, IFN*γ, *IL2/6/12A, HLA-A*, and *VEGFA*). Interestingly, there were significant increases in *Foxp3* and *IL10* expression, which are associated with immunosuppression in the tumor microenvironment.

Although the precise molecular mechanism and specific different constituents are unknown, these data suggest that malignant cells secreted exosomes in the tumor microenvironment to recruit lymphocytes not only to suppress antitumor immunity (IL6, Foxp3, and HLA-A/B) but also to enhance tumor invasion, tumor angiogenesis, and dissemination of proinflammatory cytokines (such as IL6 and VEGFA). More importantly, the immunity pathway analyses highlighted several genes with significant differences in mRNA expression in the PCR array, suggesting that the interferon and NF-κB signaling pathways are involved in the immune regulation of malignant exosomes. Owing to the complex nature of cancer and individual differences in the immune system, further studies with larger cohorts are warranted to confirm these results and determine key immunological biomarkers in the tumor microenvironment that may help develop better immunotherapeutic strategies.

## Ethics Statement

The Ethics Committee of Nanjing Medical University. All research involving human participants was conducted with approval from the Ethics Committee of Nanjing Medical University. Written informed consent was obtained from all participants. None of the patients were treated with preoperative chemotherapy or radiation. No these additional considerations in the study.

## Author Contributions

YL and YY participated in the design of experiments, carried out the molecular analysis of cells, interpretation and analysis of *in vitro* and *in vivo* data, and helped to draft the manuscript. AX, XW, JX, and SH were involved in drafting the manuscript and participated in all experiments involving animals, including histological analyses. SZ have been involved in all aspects of the study, including experimental design, analysis and interpretation of data, and manuscript writing. All authors read and approved the final manuscript.

## Conflict of Interest Statement

The authors declare that the research was conducted in the absence of any commercial or financial relationships that could be construed as a potential conflict of interest.
